# Dynamic changes of bone metastasis predict bone‐predominant status to benefit from radium‐223 dichloride for patients with castration‐resistant prostate cancer

**DOI:** 10.1002/cam4.3459

**Published:** 2020-09-22

**Authors:** Kohei Hashimoto, Yasuhide Miyoshi, Tetsuya Shindo, Masakazu Hori, Yasumasa Tsuboi, Ko Kobayashi, Fumimasa Fukuta, Toshiaki Tanaka, Shintaro Miyamoto, Takeshi Maehana, Manabu Okada, Naotaka Nishiyama, Masahiro Yanase, Ryuichi Kato, Hiroshi Hotta, Yasuharu Kunishima, Atsushi Takahashi, Shiro Hinotsu, Koh‐ichi Sakata, Hiroshi Kitamura, Hiroji Uemura, Naoya Masumori

**Affiliations:** ^1^ Department of Urology Sapporo Medical University School of Medicine Sapporo Japan; ^2^ Department of Urology and Renal Transplantation Yokohama City University Medical Center Yokohama Japan; ^3^ Department of Radiology Sapporo Medical University School of Medicine Sapporo Japan; ^4^ Department of Urology, Graduate School of Medicine and Pharmaceutical Sciences for Research University of Toyama Toyama Japan; ^5^ Department of Urology Sunagawa City Medical Center Sunagawa Japan; ^6^ Department of Urology Muroran City General Hospital Muroran Japan; ^7^ Department of Urology Asahikawa Redcross Hospital Asahikawa Japan; ^8^ Department of Urology Hakodate Goryoukaku Hospital Hakodate Japan; ^9^ Department of Biostatistics Sapporo Medical University School of Medicine Sapporo Japan

**Keywords:** bone‐predominant, castration‐resistant prostate cancer, prognosis, PSA doubling time, radium‐223 dichloride, risk assessment

## Abstract

**Background:**

To best employ radium‐223 dichloride (Ra‐223) for patients with castration‐resistant prostate cancer (CRPC) and bone metastasis, we investigated the bone‐predominant status in patients treated with Ra‐223.

**Methods:**

We retrospectively evaluated 127 CRPC patients who underwent treatment with Ra‐223. The patients were divided into three groups based on the types of dynamic changes of bone metastasis between diagnosis and just before Ra‐223: (a) only known lesions; (b) de novo lesions; (c) new progressive lesions. We developed the risk assessment using predictive factors based on progression‐free survival (PFS).

**Results:**

During the median follow‐up period of 10.4 months, the median PFS in the only known lesions group was 11.3 months compared to 8.1 months in the de novo lesions group and 5.1 months in the new progressive lesions group (*P* < .001). In multivariate analysis, the type of the new progressive lesions in bone metastasis (HR 1.45, 95% CI 1.13‐1.66, *P* = .003), performance status of >1 (HR 1.74, 95% CI 1.04‐2.89, *P* = .034), PSA value of >100 ng/mL (HR 1.59, 95% CI 1.02‐2.50, *P* = .043), and PSA doubling time (PSADT) of <3 months (HR 1.53, 95% CI 1.11‐2.03, *P* = .007) were independent unfavorable predictive factors for PFS. The risk assessment for PFS was highlighted when the type of dynamic changes of bone metastasis was combined with PSADT just before Ra‐223 treatment. This was associated with non‐bone metastasis progression, especially visceral metastasis, and overall survival.

**Conclusions:**

Risk assessment in combination with dynamic changes of bone metastasis and PSADT determines the bone‐predominant metastasis type to benefit from Ra‐223.

## Graphical abstract

We developed the risk assessment based on the types of dynamic changes of bone metastasis before the use of radium‐223 dichloride (Ra‐223). This risk assessment can be used to maximize the clinical benefits of Ra‐223 treatment for bone‐predominant metastasis.

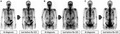

## 1 INTRODUCTION

Metastatic castration‐resistant prostate cancer (mCRPC) metastasizes to bone in up to 90% of patients with this disease.^1^ Patients with bone metastasis are vulnerable to substantial morbidity including bone pain and skeletal‐related events (SREs), which leads to worsening quality‐of‐life (QOL) and survival.^2^ This underlines the importance of management for bone metastasis in those patients. However, palliative irradiation and bone‐modifying agents, including zoledronic acid and denosumab, have not been shown to improve survival, and the benefits from these treatments are primarily limited to pain relief and delay of SREs.^3^, ^4^

Radium‐223 dichloride (Ra‐223) is a targeted alpha emitter that selectively binds to the site of high bone turnover caused by bone metastasis.^5^ Alpha particles with a very short range (<100 μm) induce predominantly double‐stranded DNA breaks that result in highly localized cytotoxic effects and less damage to the surrounding tissues. The efficacy and safety of Ra‐223 in mCRPC patients with symptomatic bone metastasis were demonstrated in the ALSYMPCA trial.^6^ With six cycles of Ra‐223 plus best standard of care (BSoC) vs placebo plus BSoC, Ra‐223 showed an overall survival benefit of 3.6 months (median 14.9 vs 11.3 months; hazard ratio [HR]), 0.70; 95% confidence interval [CI], 0.58‐0.83). The time to the first symptomatic skeletal event and QOL was also improved by Ra‐223, with a low incidence of grade 3 or 4 adverse events.^7^

Although Ra‐223 can play a major role in the management of mCRPC and symptomatic bone metastasis, it remains unclear how to best employ this treatment in clinical practice.^8^, ^9^ Some patients have disease progression of non‐bone metastasis with eventually elevated PSA levels during Ra‐223 treatment, which can occasionally be too late for the patient to receive subsequent therapies. Thus, the diagnostic certainty of bone‐predominant mCRPC needs to be determined. We hypothesized that the types of dynamic changes of bone metastasis would reflect the differential bone‐predominant status in CRPC. The aim of this study was to clarify which dynamic changes of bone metastasis before the use of Ra‐223 maximized its clinical benefits.

## 2 MATERIALS AND METHODS

This study was retrospectively conducted using the clinical data of 127 consecutive CRPC patients treated with Ra‐223 at multiple centers from May 2012 to August 2019. Approval of each study center's institutional review board was obtained for this study. All patients were diagnosed with prostate cancer by pathological examination. CRPC was defined by disease progression despite castrate levels of serum testosterone (<50 ng/dL). Bone single‐photon emission computed tomography/computed tomography (SPECT/CT) after injection of 99mTc‐methylene diphosphonate or 99mTc‐hydroxymethylene diphosphonate (bone scintigraphy), and computed tomography (CT), were conducted to identify bone metastases and soft tissue, respectively. All patients had two or more bone metastases (symptomatic or asymptomatic), no lymph node metastasis of more than 3 cm, and no visceral metastasis.

Patients received Ra‐223, 55 kBq/kg, every 4 weeks for up to six cycles and were allowed to receive concomitant BSoC, including external beam radiotherapy, corticosteroids, and first‐generation anti‐androgens such as bicalutamide and flutamide. During the Ra‐223 treatment period, chemotherapy such as docetaxel and cabazitaxel, estrogen agents, and androgen receptor‐targeted agents such as abiraterone and enzalutamide were not used.

**Figure 1:**
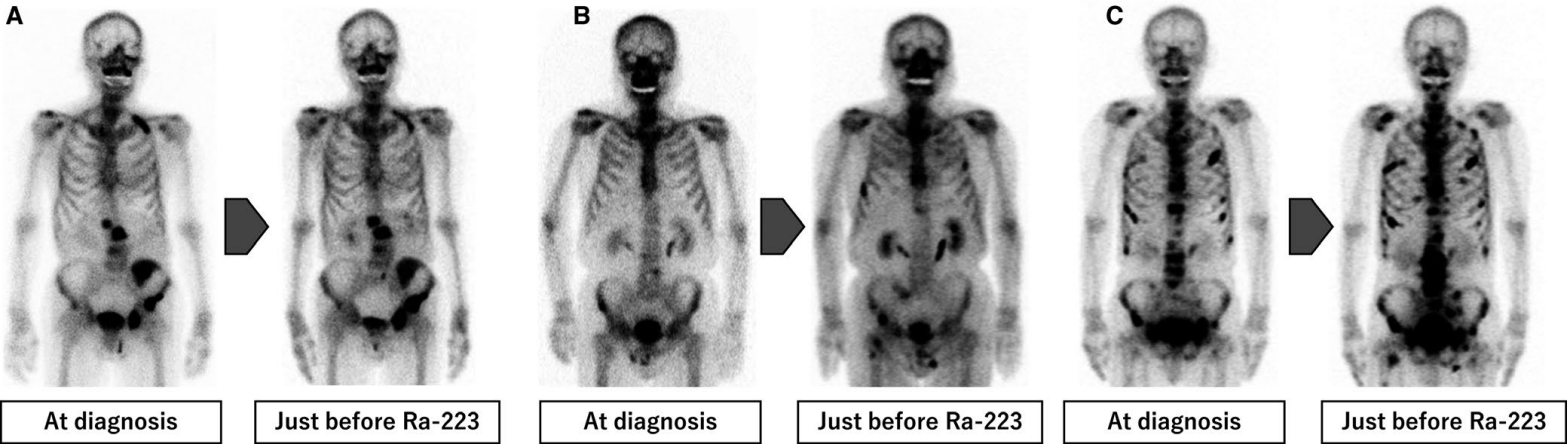
The types of dynamic changes of bone metastasis between the diagnosis of prostate cancer and just before radium‐223 dichloride (Ra‐223). Representative cases are shown. A, Only known lesions with progression, B, De novo lesions, C, New progressive lesions with the known lesions

All patients were divided into three groups by the types of radiographical dynamic changes of bone metastasis between the diagnosis of prostate cancer and just before treatment with Ra‐223: (a) only known lesions; (b) de novo lesions; (c) new progressive lesions. Representative cases are shown in Figure 1. Only known lesions were defined as bone metastasis just before Ra‐223 treatment observed in only the same lesions that progressed, improved or were not changed compared to the lesions at the diagnosis of prostate cancer. De novo lesions were defined as bone metastasis just before treatment with Ra‐223 newly diagnosed at CRPC. New progressive lesions observed in the newly developed lesions in addition to the lesions at the diagnosis of prostate cancer were defined as bone metastases just before Ra‐223 treatment. Bone scans were evaluated by qualitative and quantitative analyses using a computer‐aided diagnostic system (BONENAVI^®^, FUJIFILM Toyama Chemical Co. Ltd.). The degree of the bone tumor burden was measured by the bone scan index (BSI).^10^ In the case of bone superscans, progression was evaluated by CT findings, which were supported by calculating the mean uptake obtained as the standardized uptake value (SUV) in the trunk using bone SPECT/CT (GIBONE^®^, Nihon Medi‐Physics Co. Ltd.; Figure S1). The area above SUV = 7.0 was considered to be the active bone metastatic burden. Then the average SUVpeak was obtained as the average of the peaks of the regional SUV.^11^ When osteosclerotic lesions on bone superscans extended on CT and SUVpeak increased on bone SPECT/CT, the dynamic changes of bone metastases were classified as the new progressive lesions group. When osteosclerotic lesions on bone superscans extended without a SUVpeak increase, or osteosclerotic lesions reduced regardless of SUVpeak changes, the dynamic changes of bone metastases were classified into the only known lesions group.

PSA doubling time was calculated by PSA elevation from 3 months before Ra‐223 treatment. PSA progression was defined as per the criteria of the Prostate Cancer Working Group 3; that is, a 25% increase from the nadir (consisting of a starting value of ≥1.0 ng/mL) with a minimum rise of 2 ng/mL.^12^ A PSA response was defined as a PSA decrease of ≥50% at any time. All patients were followed‐up by PSA measurement every 1 month. CT and bone scintigraphy were conducted after Ra‐223 treatment or at need.

Fisher's exact test, the *χ* ^2^ test, and the Kruskal‐Wallis test were used as appropriate to evaluate group differences. Progression‐free survival (PFS) was defined as the time from the start of Ra‐223 treatment to the time of PSA progression during the first subsequent therapy, clinical/radiographical progression or death from any cause, whichever occurred first. The cumulative incidence of bone metastasis progression events (bPEs) was defined as the probability that the first evidence of bone metastatic progression such as new lesions, extension of known lesions or the last tumor evaluation occurred after the initiation of Ra‐223 treatment. The cumulative incidence of non‐bone metastasis progression events (nbPEs) was defined as the probability of the first evidence of visceral metastasis, nodal metastatic progression or primary local progression, whichever occurred first after the initiation of Ra‐223 treatment. The Fine–Gray regression model was used to calculate the probability of bPEs or nbPEs. Cumulative incidence curves were used in a competing risk setting, with death without bPEs or nbPEs for the probability of bPEs or nbPEs as a competing event. These were estimated until 3 years because of the number of patients at risk. PFS and overall survival (OS) were evaluated using the Kaplan–Meier method, with the log‐rank test used to evaluate differences between the groups. Multivariate Cox proportional hazards analysis was used to identify factors to predict PFS of Ra‐223 and included all factors that were significantly associated with a univariate analysis model. Based on the HR for PFS, we developed a risk model with three categories: favorable, intermediate, and poor. A *P*‐value of <.05 was considered statistically significant for all analyses. All statistical analyses were performed using EZR (Jichi Medical University, Saitama, Japan).

## 3 RESULTS

The characteristics of the patients with the various types of dynamic changes of bone metastasis were compared (Table 1). A higher proportion of patients in the type with de novo lesions had a long time to CRPC compared with other types because of a less advanced T stage at diagnosis and undergoing prior local therapy. Patients with new progressive lesions had a higher PSA value and higher ALP value just before Ra‐223 treatment than those with the other types. There were no significant differences in bone pain or the extent of disease among the types of bone metastasis.

**Table 1:** Characteristics of patients according to the types of dynamic changes of bone metastasis

	Overall n = 127	Dynamic changes of bone metastasis			*P* value
		Only known n = 66	De novo n = 26	New progressive n = 35	
Age, y, median (IQR)	75 (69‐79)	73 (66‐78)	76 (72‐82)	76 (70‐80)	.102
ECOG PS, n (%)					.207
0	45 (35)	26 (39)	8 (31)	11 (31)	
1	58 (46)	32 (49)	13 (50)	13 (37)	
≥2	24 (19)	8 (12)	5 (19)	11 (31)	
Gleason score, n (%)					.36
≤7	9 (7)	2 (3)	4 (15)	3 (9)	
8	45 (36)	24 (38)	9 (35)	12 (35)	
≥9	70 (57)	38 (59)	13 (50)	19 (56)	
Unclear	3	2		1	
T stage at diagnosis, n (%)					.004
≤T2	27 (21)	8 (12)	13 (50)	6 (17)	
T3	73 (58)	42 (64)	11 (42)	20 (57)	
T4	27 (21)	16 (24)	2 (8)	9 (26)	
Time to CRPC, mo, median (IQR)	14.1 (6.3‐32.2)	11.4 (5.7‐18.4)	34.5 (15.7‐67.3)	15.4 (7.6‐34.8)	<.001
Bone pain at metastasis, n (%)	61 (48)	26 (39)	16 (62)	19 (54)	.095
PSA, ng/mL, median (IQR)	45.8 (10.2‐243)	23.2 (6.8‐95.3)	45.9 (9.9‐197)	130 (45.0‐437)	.002
ALP, U/L, median (IQR)	324 (225‐534)	273 (197‐459)	325 (259‐414)	438 (303‐971)	.045
PSA doubling time, mo, median (IQR)	2.4 (1.3‐3.8)	2.6 (1.4‐5.3)	2.5 (1.2‐3.2)	2.4 (1.4‐3.0)	.214
Lymph node metastasis, n (%)	22 (17)	12 (18)	3 (12)	7 (20)	.707
Extent of disease, n (%)					.067
≤5	38 (30)	23 (35)	8 (31)	7 (20)	
6‐20	39 (31)	19 (29)	12 (46)	8 (23)	
≥21	38 (30)	19 (29)	6 (23)	13 (37)	
Superscans	12 (9)	5 (7)	0	7 (20)	
Concurrent use of zoledronate or denosumab, n (%)	86 (68)	47 (71)	14 (54)	25 (71)	.237
Prior local treatment, n (%)	21 (16)	5 (7)	14 (54)	2 (6)	<.001
Prior treatment in CRPC, n (%)					.83
Docetaxel	58 (45)	25 (38)	12 (46)	21 (60)	
Abiraterone	59 (46)	26 (39)	12 (46)	21 (60)	
Enzalutamide	48 (38)	18 (27)	14 (54)	16 (46)	
Numbers of prior treatments, n (%)					.055
≤2	69 (55)	43 (65)	14 (54)	12 (34)	
3	22 (17)	9 (14)	5 (19)	8 (23)	
≥4	36 (28)	14 (21)	7 (27)	15 (43)	

Note

Variables are expressed as the median (interquartile range: IQR) or n (%).

Abbreviations: ALP, alkaline phosphatase; CRPC, castration‐resistant prostate cancer; ECOG PS, Eastern Cooperative Oncology Group performance status; PSA, prostate‐specific antigen.

**Figure 2:**
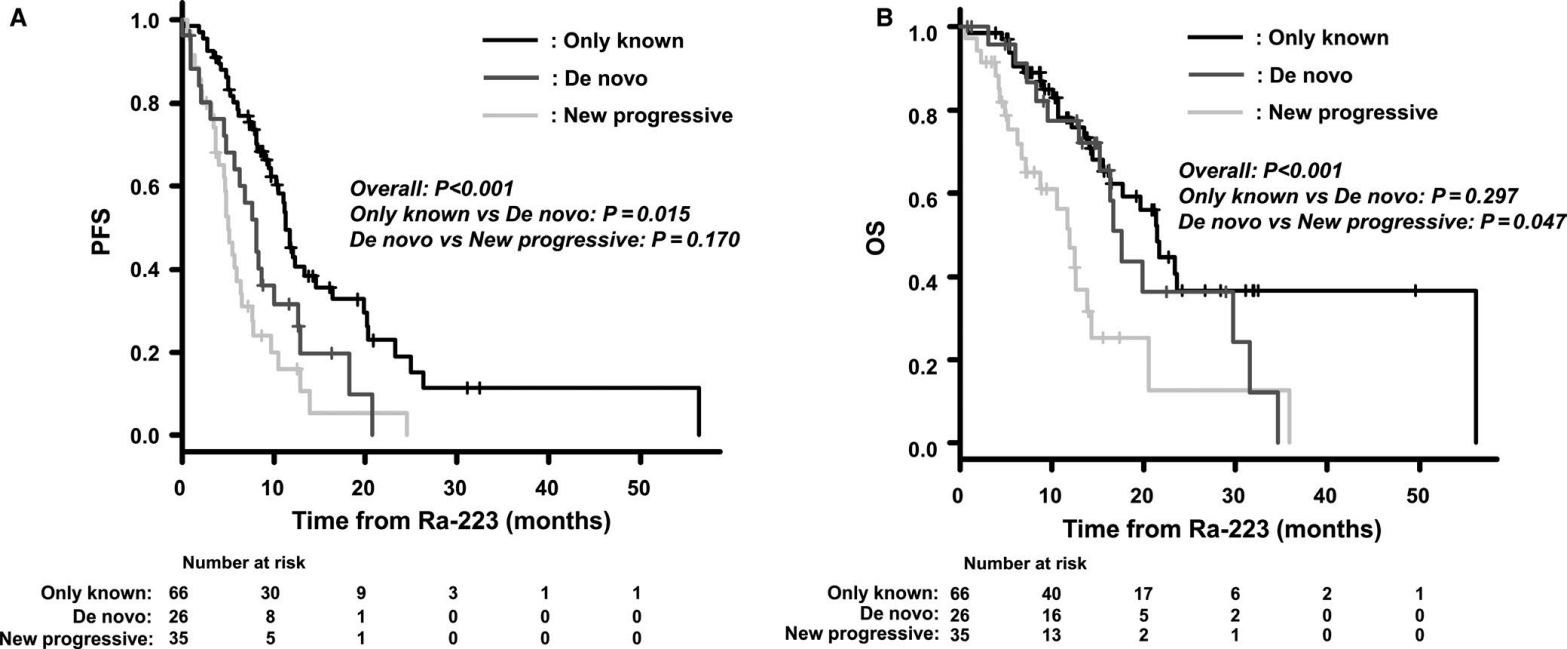
Kaplan–Meier probability curves according to the type of dynamic changes of bone metastasis before treatment with radium‐223 dichloride (Ra‐223). A, progression‐free survival (PFS), B, overall survival (OS)

PSA responses were observed in 11 patients (9%) and ALP declines of >30% in 52 (41%). During the median follow‐up period of 10.4 months, 58 patients (46%) died of prostate cancer and three (2%) died from other causes. The median PFS, bPEs, nbPEs, and OS were 8.1, 14.4, 5.7, and 17.7 months, respectively. Patients in the only known lesions group were more likely to have Ra‐223 of ≥5 cycles and six cycles compared to those with de novo lesions and new progressive lesions (83%, 65% vs 66%; *P* = .070, 77%, 57% vs 43%; *P* = .002, respectively; Table S1). There were significant differences in PFS and OS among the types of dynamic changes of bone metastasis (Figure 2A,B). The median PFS and OS in the only known lesions group were 11.3 and 21.5 months, respectively, compared to 8.1 and 17.7 months in the de novo lesions group and 5.1 and 11.9 months, respectively, in the new progressive lesions group (*P* < .001). Univariate analysis revealed that the types of dynamic changes of bone metastasis, Eastern Cooperative Oncology Group performance status (PS), bone pain, PSA value, ALP value, PSADT, and prior use of docetaxel were associated with PFS (Table 2). The number of prior treatments was excluded from the variables because it was a confounder to prior use of docetaxel (*P* = .001). In multivariate analysis, new progressive lesions in bone metastasis (HR 1.45, 95% CI 1.13‐1.66, *P* = .003), PS of >1 (HR 1.74, 95% CI 1.04‐2.89, *P* = .034), a PSA value of >100 ng/mL (HR 1.59, 95% CI 1.02‐2.50, *P* = .043), and PSADT of <3 months (HR 1.53, 95% CI 1.11‐2.03, *P* = .007) were independent unfavorable predictive factors for PFS.

**Table 2:** Factors predicting disease progression during subsequent therapy in patients treated with Ra‐223

Variable	Category	Univariate		Multivariate	
		HR (95% CI)	*P* value	HR (95% CI)	*P* value
Dynamic changes of bone metastasis	New progressive (Ref: Only known, De novo)	1.47 (1.15‐1.86)	<.001	1.45 (1.13‐1.66)	.003
Age, y	≥80 (Ref: <80)	1.32 (0.81‐2.15)	.269		
ECOG PS	≥2 (Ref: <2)	2.00 (1.22‐3.27)	.006	1.74 (1.04‐2.89)	.034
Gleason score	≥9 (Ref: ≤8)	1.12 (0.80‐1.56)	.503		
T stage at diagnosis	≥T3b (Ref: ≤T3a)	1.12 (0.74‐1.72)	.586		
Time to CRPC, mo	<12 (Ref: ≥12)	1.25 (0.83‐1.89)	.289		
Bone pain at metastasis	Yes (Ref: No)	1.26 (1.15‐2.16)	.032	1.13 (0.69‐1.71)	.092
PSA, ng/mL	≥100 (Ref: <100)	2.00 (1.31‐3.06)	.001	1.59 (1.02‐2.50)	.043
ALP, U/L	>ULN (Ref: ≤ULN)	1.67 (1.11‐2.54)	.015	1.22 (0.87‐1.89)	.087
PSA doubling time, mo	<3 (Ref: ≥3)	1.57 (1.17‐2.10)	.002	1.53 (1.11‐2.03)	.007
Lymph node metastasis	Yes (Ref: No)	0.54 (0.24‐1.49)	.251		
Extent of disease	Superscans (Ref: <superscans)	1.13 (0.56‐2.25)	.736		
Concurrent use of zoledronate or denosumab	No (Ref: Yes)	1.10 (0.84‐1.37)	.583		
Prior docetaxel	Yes (Ref: No)	1.68 (1.11‐2.54)	.015	1.34 (0.85‐2.10)	.205

Abbreviations: ALP, alkaline phosphatase; CRPC, castration‐resistant prostate cancer; ECOG PS, Eastern Cooperative Oncology Group performance status; HR, hazard ratio; PSA, prostate‐specific antigen; Ra‐223, radium‐223 dichloride; Ref, referent; ULN, upper limit of normal.

**Figure 3:**
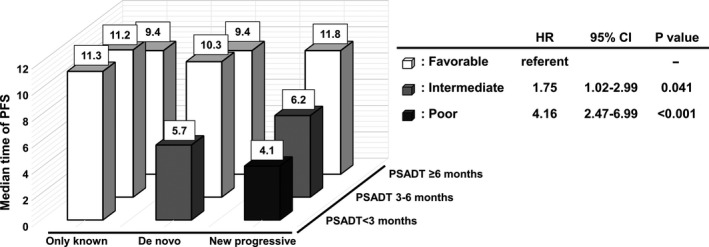
The risk assessment of progression‐free survival (PFS) in combination with the dynamic changes of bone metastasis by type and PSA doubling time (PSADT) just before radium‐223 dichloride (Ra‐223) treatment (white cuboid, favorable; gray cuboid, intermediate; black cuboid, poor). A, median time of PFS, B, hazard ratio (HR) according to the risk category

To clarify the characteristics of bone metastasis maximizing the benefit of Ra‐223 in patients with bone metastasis, we developed a risk model based on PFS in combination with dynamic changes of bone metastasis by type and PSADT just before treatment with Ra‐223 (Figure 3). Favorable risks were defined as only known lesions and any PSADT, de novo lesions and PSADT of ≥3 months, and any new progressive lesions and PSADT of ≥6 months, whereas intermediate risks were de novo lesions and PSADT of <3 months, or any new progressive lesions and PSADT of 3 to <6 months, and poor risk any new progressive lesions and PSADT of <3 months. The HR for PFS in those with intermediate risk and poor risk was high compared to that for the favorable risk group (HR 1.75, 95% CI 1.02‐2.99; *P* = .041, and HR 4.16, 95% CI 2.47‐6.99; *P* < .001, respectively).

The PSA response, ALP value, and completion of Ra‐223 were assessed for the risk groups (Table 3). A PSA response was observed in 11 patients (13%) in the favorable risk group, but in none in the intermediate risk and poor risk groups (*P* = .046). There was no significant difference in the decline in ALP levels among the risk groups. Completion of Ra‐223 treatment was achieved in 64 patients (77%) in the favorable risk group, 10 (48%) in the intermediate risk group, and 7 (30%) in the poor risk group (*P* < .001). Patients with a favorable risk or intermediate risk were more likely to have a PSADT of ≥6 months during Ra‐223 treatment even if they had a PSADT of <3 months just before it (Figure S2). Patients in the poor risk group were more likely to have the best supportive care as sequential therapy.

**Table 3:** Response during Ra‐223 treatment and the kinds of subsequent therapy according to the risk category

Risk category	Favorable n = 83	Intermediate n = 21	Poor n = 23	*P* value
PSA decline, n (%)				
Any	17 (20)	2 (10)	0	.024
≥30%	13 (16)	1 (5)	0	.062
≥50%	11 (13)	0	0	.046
ALP decline, n (%)				
≥30%	33 (40)	9 (43)	9 (39)	.965
≥50%	27 (32)	4 (19)	6 (26)	.487
Ra‐223 cycles, n (%)				
≥5	69 (83)	14 (67)	12 (52)	.006
6	64 (77)	10 (48)	7 (30)	<.001
Subsequent therapy, n (%)				<.001
Docetaxel	20 (24)	4 (19)	2 (9)	
Abiraterone	17 (20)	2 (9)	2 (9)	
Enzalutamide	13 (16)	7 (33)	0	
Cabazitaxel	2 (2)	1 (5)	0	
BSC	18 (22)	6 (29)	17 (73)	
Others	13 (16)	1 (5)	2 (9)	

Note

Variables are expressed as n (%).

Abbreviations: ALP, alkaline phosphatase; BSC, best supportive care; PSA, prostate‐specific antigen; Ra‐223, radium‐223 dichloride.

**Figure 4:**
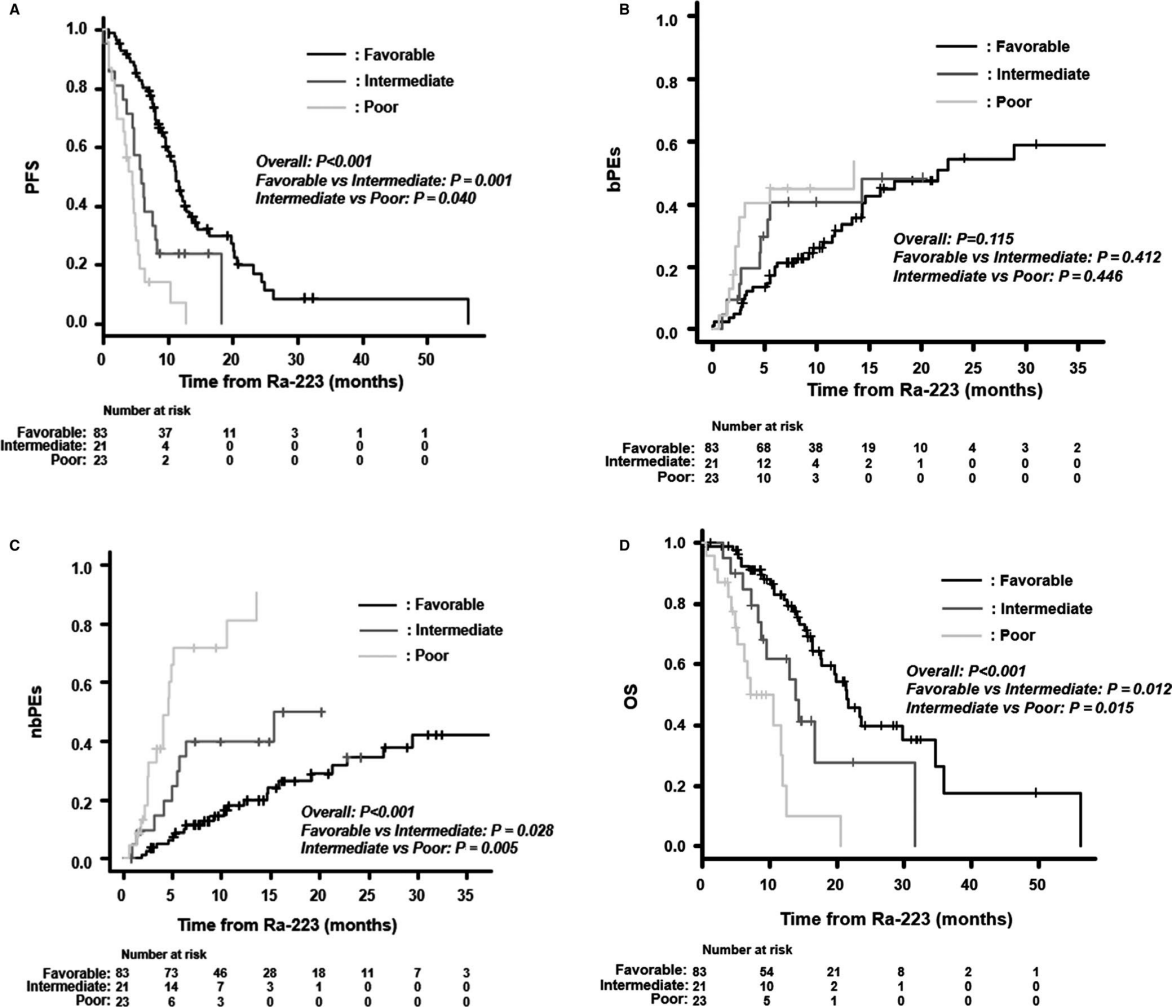
Kaplan‐Meier probability curves (A, D) and Fine–Gray incidence curves (B, C) according to the risk category: A, progression‐free survival (PFS), B, bone metastasis progression events (bPEs), C, non‐bone metastasis progression events (nbPEs), D, overall survival (OS)

We assessed PFS, bPEs, nbPEs, and OS according to the risk group. PFS was significantly longer in the favorable risk group than in the intermediate and poor risk groups (median, 11.3 vs 5.9 vs 4.6 months, *P* < .001; Figure 4A). In the poor risk group, the 1‐year bPEs rate was 45% compared to 32% in the favorable risk group and 41% in the intermediate risk group (*P* = .115), while the 1‐year nbPEs rate was 81% in the poor risk group compared to 18% in the favorable risk group and 40% in the intermediate risk group (*P* < .001; Figure 4B,C). Fifteen (94%) of the 16 patients in the poor risk group who had non‐bone metastasis progression developed visceral metastasis. The median OS in the favorable risk group was 21.5 months compared to 13.8 months in the intermediate risk group and 7.2 months in the poor risk group (*P* < .001; Figure 4D).

## 4 DISCUSSION

We developed a risk assessment model to determine the bone‐predominant metastasis type in CRPC patients that will benefit from Ra‐223 treatment. The dynamic changes of bone metastasis, PSADT, PSA just before Ra‐223 treatment, and PS were significantly associated with PFS in patients treated with Ra‐223. In particular, the dynamic changes of bone metastasis combined with PSADT highlighted the optimal decision for the use of Ra‐223. The favorable risks, defined as only known lesions and any PSADT, de novo lesions, and PSADT of ≥3 months, or any new progressive lesions and PSADT of ≥6 months, were associated with better nbPEs and OS as well. In contrast, the poor risk, defined as any new progressive lesions and PSADT of <3 months, was associated with poor nbPEs and OS. Those with the poor risk were presumably more likely to have the risk of progression of visceral metastasis following Ra‐223 treatment. This risk assessment may help to determine the ideal sequencing of other life‐prolonging therapies following Ra‐223 treatment.

In several studies, Ra‐223 was found to confer a survival benefit even after docetaxel therapy.^13^, ^14^ However, there may still be room for improvement regarding the timing for the use of Ra‐223 in real clinical practice because some patients have an early cessation of Ra‐223 due to death or disease progression. Recent studies have shown that patients with 1‐4 doses of Ra‐223 are more likely to have shorter OS than those with 5‐6 doses.^15^, ^16^, ^17^, ^18^, ^19^ The cessation of Ra‐223 was associated with poor PS, severe pain, and a higher PSA level at baseline and a lower hemoglobin level at baseline. Likewise, the present study demonstrated that patients with PSA values of >100 ng/mL at baseline and PS ≥2 were more likely to have worse PFS with Ra‐223. Thus, it appears to be clear that earlier use of Ra‐223, when the patient has a lesser disease burden than indicated by these poor baseline factors, should be considered. These findings are supported by the international early access program (iEAP) findings that mCRPC patients with no symptoms had better OS than those with symptoms.^20^

Another concern is the occasional loss of the opportunity to receive subsequent life‐prolonging therapies even if the patient is treated with a full schedule of Ra‐223. We must avoid a treatment period of Ra‐223 that sacrifices the remaining time of patients due to the uncontrolled progression of non‐bone metastasis. In the present study, 41 of the patients (32%) had no choice but best supportive care following Ra‐223 treatment in spite of rates of PSA responses similar to those in other reports.^6^, ^21^ Of those, 21 had two or more choices for life‐prolonging therapies before the initiation of Ra‐223 treatment. This indicates that we could not have identified bone‐predominant mCRPC in some patients treated with Ra‐223. A recent new imaging study showed the utility of gallium Ga 68‐labeled positron emission tomography tracer targeting of prostate‐specific membrane antigen (68Ga‐PSMA‐11 PET) for planning Ra‐223 treatment.^22^ In the study, patients selected by PSMA PET and bone scintigraphy were more likely to have a PSA decline (44%) with Ra‐223 treatment. Therefore, in addition to the completion of the Ra‐223 course, which patients will benefit most from Ra‐223 should be determined for bone‐predominant mCRPC to best address the following treatment with maintenance for control of bone metastasis.

Assessment discriminating progression of bone and/or non‐bone metastasis is needed to clarify bone‐predominant mCRPC. We focused on dynamic changes of bone metastasis having differential biological progression. The risk assessment using dynamic changes of bone metastasis by type was highlighted by the PSADT just before Ra‐223 treatment, which was one of the significant factors associated with PFS, because PSADT just before Ra‐223 well reflected the biological dynamics in the progression of bone and/or non‐bone metastasis. This risk assessment consisting of the dynamic status of bone metastasis by the type and PSADT just before Ra‐223 treatment was significantly associated with the rate of the PSA response and the completion rate of Ra‐223 treatment, which was supported by nbPEs, OS, and the proportion of best supportive care in the first subsequent therapy. Actually, patients with a favorable risk were more likely to have a slow elevation of the PSA level even if the value was increased during Ra‐223 treatment, whereas those with the poor risk were less likely to have one. The decline in the ALP level was similar to those in other reports and was steadily observed regardless of the risk group.^6^, ^21^ It is likely that Ra‐223 exerted a biological effect on bone metastasis even in those with the poor risk, but its duration was limited based on the results of bPEs in the present study. This risk assessment clearly appeared to predict bone‐predominant mCRPC in that there were significant differences in PFS and nbPEs among the risk groups.

The limitations of our study should be acknowledged. Foremost are the retrospective design of our study, the short follow‐up period, and the small number of patients. Dynamic radiographical changes of bone metastases were evaluated between the diagnosis of prostate cancer, not CRPC, and just before treatment with Ra‐223 because of the various treatment lines of Ra‐223. These might have substantially affected the results. Moreover, the efficacy of Ra‐223 alone was insufficient for patients with the intermediate risk. For such patients, it remains challenging to determine whether combination therapy can be effective and safe. In the ERA 223 trial, a double‐blind, randomized, placebo‐controlled, phase 3 study, concurrent treatment with Ra‐223, and abiraterone acetate plus prednisone or prednisolone did not improve symptomatic skeletal event‐free survival or OS in chemotherapy‐naive mCRPC patients with bone metastasis.^23^ In that study, there was a higher incidence of bone fracture in the Ra‐223 and abiraterone arm. The issue of concurrent treatment with Ra‐223 and abiraterone acetate plus prednisone or prednisolone inducing extremely poor bone health has been discussed in terms of several findings such as obvious depletion of testosterone and its metabolite, estrogen, and inhibition of osteoblast activity in the healthy bone microenvironment.^24^ Concurrent use of a bone‐mediating agent such as zoledronate or denosumab strongly appeared to be important in Ra‐223 treatment. Another phase 3 trial (PEACE III, NCT02194842) with Ra‐223 and a placebo, each in combination with enzalutamide, is ongoing. Further study is needed to clarify the benefit of Ra‐223 in combination with other agents involved in the setting of each treatment line for CRPC.

## 5 CONCLUSIONS

We have shown that the type of dynamic changes of bone metastasis is useful to determine the indication for Ra‐223 treatment in patients with mCRPC. The risk assessment for PFS was highlighted when the type of dynamic changes of bone metastasis was combined with PSADT just before the use of Ra‐223. Patients with the favorable risk had a high proportion of completion of Ra‐223 treatment and a decline or a slow elevation in the PSA level during the treatment with Ra‐223. They had better nbPEs and OS. Our findings suggest that this risk assessment can be used to maximize the clinical benefits of Ra‐223 treatment for bone‐predominant metastasis, which may make possible ideal sequencing of other life‐prolonging therapies following the use of Ra‐223.

## CONFLICT OF INTEREST

No potential conflict of interest was reported by the authors.

## AUTHOR CONTRIBUTIONS

Kohei Hashimoto: Conceptualization; data curation, investigation, methodology, project administration, formal analysis, and writing. Yasuhide Miyoshi: Investigation, data curation, and project administration. Tetsuya Shindo: Investigation, data curation, and project administration. Masakazu Hori: Project administration. Yasumasa Tsuboi: Data acquisition. Ko Kobayashi: Investigation and data curation. Fumimasa Fukuta: Investigation and data curation. Toshiaki Tanaka: Investigation and data curation. Shintaro Miyamoto: Data acquisition. Takeshi Maehana: Data acquisition. Manabu Okada: Data acquisition. Naotaka Nishiyama: Data acquisition. Masahiro Yanase: Investigation. Ryuichi Kato: Investigation. Hiroshi Hotta: Investigation. Yasuharu Kunishima: Investigation. Atsushi Takahashi: Investigation and project administration. Shiro Hinotsu: Formal analysis and project administration. Koh‐ichi Sakata: Project administration. Hiroshi Kitamura: Investigation, data curation, and project administration. Hiroji Uemura: Investigation and project administration. Naoya Masumori: Conceptualization, methodology, writing, and supervision.

## Supporting information

Fig S1

Fig S2

Table S1

## Data Availability

The data that support the findings of this study are available from the corresponding author, NM, upon reasonable request.
