# Supplementary figures and images for: Dynamic changes of bone metastasis predict bone‐predominant status to benefit from radium‐223 dichloride for patients with castration‐resistant prostate cancer
Source: Cancer Med. 2020 Sep 22;9(22):8579–88. doi: 10.1002/cam4.3459 (PMC7666734; doi:10.1002/cam4.3459)

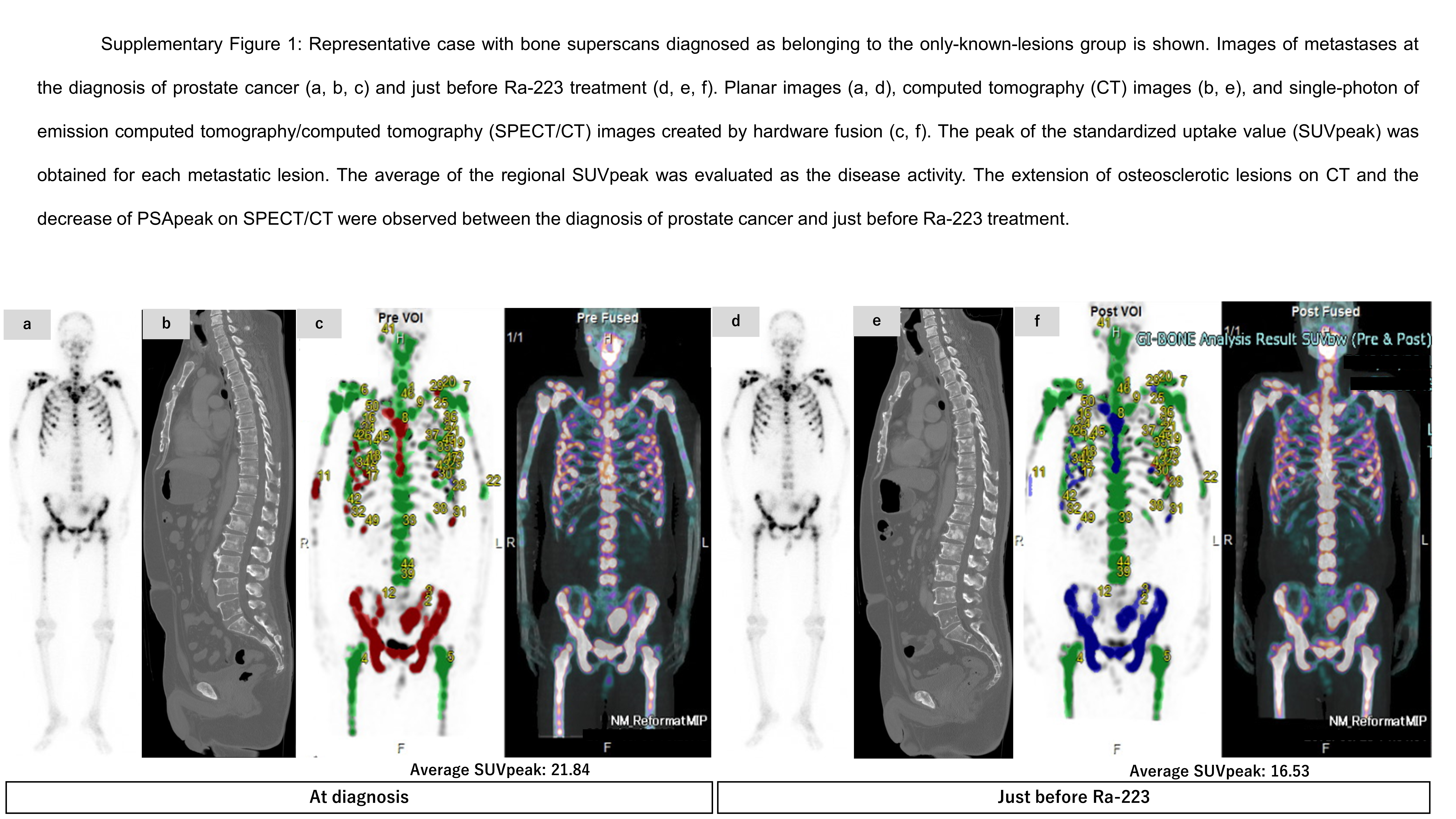

Supplement: Supplementary file 1 — Fig S1 [file CAM4-9-8579-s001.TIF]

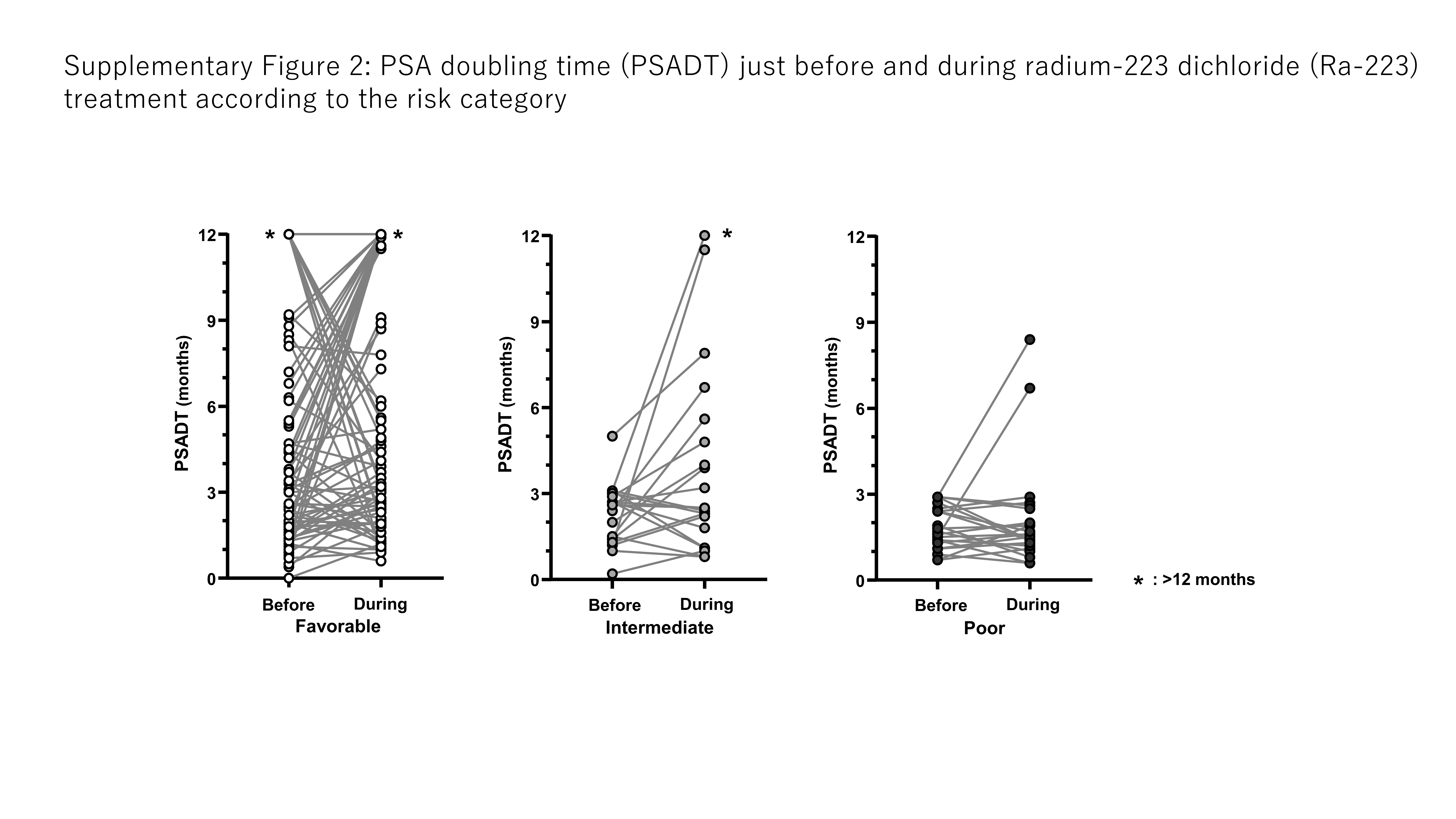

Supplement: Supplementary file 2 — Fig S2 [file CAM4-9-8579-s002.TIF]
