# Supplementary material for: Dynamic changes of bone metastasis predict bone‐predominant status to benefit from radium‐223 dichloride for patients with castration‐resistant prostate cancer
Source: Cancer Med. 2020 Sep 22;9(22):8579–88. doi: 10.1002/cam4.3459 (PMC7666734; doi:10.1002/cam4.3459)
Supplement: Supplementary file 3 — Table S1 [file CAM4-9-8579-s003.docx]

**Supplementary Table S1. Number of Ra-223 cycles according to the type of dynamic changes of bone metastasis**

|  | Overall  n=127 | Dynamic changes of bone metastasis | | | | P value |
| --- | --- | --- | --- | --- | --- | --- |
|  |  | Only known  n=66 | | De novo  n=26 | New progressive  n=35 |  |
| Numbers of Ra-223 cycles, n (%)  1  2  3  4  5  6 | 5(4)  9(7)  10(8)  8(6)  14(11)  81(64) | 2(3)  1(2)  4(6)  4(6)  4(6)  51(77) | 2(8)  4(15)  2(8)  1(4)  2(8)  15(57) | | 1(3)  4(11)  4(11)  3(9)  8(23)  15(43) | 0.034 |

Abbreviations: Ra-223, radium-223 dichloride.
